# Dual-task versus single-task gait rehabilitation after stroke: the protocol of the cognitive-motor synergy multicenter, randomized, controlled superiority trial (SYNCOMOT)

**DOI:** 10.1186/s13063-023-07138-x

**Published:** 2023-03-08

**Authors:** Sophie Tasseel-Ponche, Martine Roussel, Monica N. Toba, Thibaud Sader, Vincent Barbier, Arnaud Delafontaine, Jonathan Meynier, Carl Picard, Jean-Marc Constans, Alexis Schnitzler, Olivier Godefroy, Alain Pierre Yelnik

**Affiliations:** 1 Department of Physical Medicine and Rehabilitation, Amiens University Hospital, Amiens, France; 2 Laboratory of Functional Neurosciences, UR UPJV 4559, Jules Verne University of Picardie, Amiens, France; 3 Department of Neurology, Amiens University Hospital, Amiens, France; 4 Clinical Research and Innovation Directorate, Amiens University Hospital, Amiens, France; 5 Department of Radiology, Amiens, University Hospital, Amiens, France; 6 PRM Department, Hôpital Lariboisière-F.Widal AP-HP, Paris, France; 7 INSERM U1153 - CRESS EpiAgeing, Paris University, Hôtel-Dieu, Paris, France; 8 UMR 9010, Paris University, Centre Borelli, Paris, France

**Keywords:** Stroke, Rehabilitation, Multitask, Gait, Cognitive-motor interference, Protocol, Quality of life

## Abstract

**Background:**

Gait disorders and cognitive impairments are prime causes of disability and institutionalization after stroke. We hypothesized that relative to single-task gait rehabilitation (ST GR), cognitive-motor dual-task (DT) GR initiated at the subacute stage would be associated with greater improvements in ST and DT gait, balance, and cognitive performance, personal autonomy, disability, and quality of life in the short, medium and long terms after stroke.

**Methods:**

This multicenter (*n*=12), two-arm, parallel-group, randomized (1:1), controlled clinical study is a superiority trial. With *p*<0.05, a power of 80%, and an expected loss to follow-up rate of 10%, the inclusion of 300 patients will be required to evidence a 0.1-m.s^−1^ gain in gait speed. Trial will include adult patients (18–90 years) in the subacute phase (0 to 6 months after a hemispheric stroke) and who are able to walk for 10 m (with or without a technical aid). Registered physiotherapists will deliver a standardized GR program (30 min three times a week, for 4 weeks). The GR program will comprise various DTs (phasic, executive function, praxis, memory, and spatial cognition tasks during gait) in the DT (experimental) group and gait exercises only in the ST (control) group. The primary outcome measure is gait speed 6 months after inclusion. The secondary outcomes are post-stroke impairments (National Institutes of Health Stroke Scale and the motor part of the Fugl-Meyer Assessment of the lower extremity), gait speed (10-m walking test), mobility and dynamic balance (timed up-and-go test), ST and DT cognitive function (the French adaptation of the harmonization standards neuropsychological battery, and eight cognitive-motor DTs), personal autonomy (functional independence measure), restrictions in participation (structured interview and the modified Rankin score), and health-related quality of life (on a visual analog scale). These variables will be assessed immediately after the end of the protocol (probing the short-term effect), 1 month thereafter (the medium-term effect), and 5 months thereafter (the long-term effect).

**Discussion:**

The main study limitation is the open design. The trial will focus on a new GR program applicable at various stages after stroke and during neurological disease.

**Trial registration:**

NCT03009773. Registered on January 4, 2017.

**Supplementary Information:**

The online version contains supplementary material available at 10.1186/s13063-023-07138-x.

## Administrative information

Note: the numbers in curly brackets in this protocol refer to SPIRIT checklist item numbers. The order of the items has been modified to group similar items (see http://www.equator-network.org/reporting-guidelines/spirit-2013-statement-defining-standard-protocol-items-for-clinical-trials/).

**Table:** 

Title {1}	Dual-task versus single-task gait rehabilitation after stroke: the protocol of the cognitive-motor synergy multicenter, randomized, controlled superiority trial (SYNCOMOT)
Trial registration {2a and 2b}.	NCT03009773. Registered on January 4, 2017. https://clinicaltrials.gov/ct2/show/NCT03009773 Institutional review board: CPP Nord-Ouest II, Amiens, France: reference: RCB 2016-A00342-49, dated May 13, 2016.
Protocol version {3}	V5.0 dated December 3, 2021.
Funding {4}	The study is funded by the French state through the Hospital-Based Clinical Research Program.
Author details {5a}	Sophie Tasseel-Ponche^1,2^, Martine Roussel^2,3^, Monica Toba^2^, Thibaud Sader^1^, Vincent Barbier^1^, Arnaud Delafontaine^1^, Jonathan Meynier^4^, Carl Picard^4^, Jean-Marc Constans^5^, Alexis Schnitzler^6,7^, Olivier Godefroy^2,3^_,_ Alain Pierre Yelnik^6,8^ 1 Department of Physical Medicine and Rehabilitation, Amiens University Hospital, Amiens, France 2 Laboratory of Functional Neurosciences, UR UPJV 4559, Jules Verne University of Picardie, Amiens, France 3 Department of Neurology, Amiens University Hospital, Amiens, France 4 Clinical Research and Innovation Directorate, Amiens University Hospital, Amiens, France 5 Department of Radiology, Amiens, University Hospital, Amiens, France 6 PRM Department, Hôpital Lariboisière-F.Widal AP-HP, Paris, France 7 INSERM U1153 - CRESS EpiAgeing, Paris University, Hôtel-Dieu, Paris, France 8 UMR 9010, Paris University, Centre Borelli, Paris, France
Name and contact information for the trial sponsor {5b}	The study is funded by the French state through the Hospital-Based Clinical Research Program. The study sponsor is Amiens University Medical Center (contact address: Direction de la Recherche Clinique et de l'Innovation, CHU Amiens Picardie, 1 rond-point du Pr C. Cabrol, Cedex 1, Amiens, France).
Role of sponsor {5c}	The study’s funder and sponsor had no role in the study design or the collection, analysis, interpretation and presentation of the study data.

## Introduction

### Background and rationale {6a}

Stroke is the main cause of acquired, severe disability [1, 2]. The overall burden of stroke (quantified as disability-adjusted life years) is increasing worldwide [3]. Post-stroke sensorimotor and perceptual impairments can limit gait and thus restrict community ambulation. Indeed, gait and balance complaints are expressed by 80% of survivors 3 months after the stroke [1, 4, 5]. Gait disorders constitute a key marker of recovery from stroke [6] and are significantly and independently associated with disability [7], institutionalization, and death [8–10]. This is why stroke neurorehabilitation focuses on gait and thus improvements in the patients’ survival, personal autonomy, social inclusion, and quality of life (QoL) [8, 11–13].

Although gait rehabilitation (GR) is effective for motor impairments [14–16], falls remain a major risk factor for stroke complications [16, 17]. The development of effective methods for balance and mobility rehabilitation is a priority for stroke patients, their caregivers, and healthcare professionals [18]. Although GR is often effective for gait during rehabilitation sessions, the transfer of gait skills to daily living is more challenging; this discrepancy can be due to a change in attention, which is focused on gait during the training session but not on gait in everyday life [19–21]. These observations suggest that cognitive-motor interference (CMI) is a major contributor to activities of daily living after stroke [19–21].

It is known that CMI is abnormally frequent among stroke victims [20]. Dual-task (DT) walking can be used to evaluate CMI and attention-demanding mobility functions after stroke [22, 23]. Community ambulation, social inclusion, and QoL require the ability to simultaneously walk and execute one or more cognitive tasks [9, 24–27]. Poor functional mobility after stroke appears to be linked to worsened DT ability and attentional impairments [28]. CMI can account for the greater relative deterioration in DT performance than in single task (ST) performance [29]. This DT deterioration has been attributed to the greater attentional allocation needed to compensate for the gait impairment (requiring functional executive networks) [22]. Executive functions are often impaired after a stroke, and these higher cognitive processes (e.g., volition, planning, purposive action, action monitoring, and cognitive inhibition) are required for the coordination of cortical sensory-motor systems during DT [30]. Several theoretical frameworks for CMI have been put forward [21]. Firstly, the central capacity-sharing model holds that the two tasks have to share the available processing resources; hence, performance worsens when the demand overloads the limited attentional-sharing capacity [31, 32]. Secondly, the bottleneck model holds that each task may need simultaneous access to a processor that can only act with one input at a time; hence, the processing of the second task has to be postponed [21, 33, 34]. Thirdly, the cross-talk model predicts that tasks from different networks disturb each other (through cross-talk) but that tasks from the same network do not [35].

The new SYNCOMOT (“SYNergie Cognitivo-MOTrice” in French, which means “cognitive motor synergy” in English) GR protocol addresses the CMI problem by seeking to leverage cognitive-motor synergy in the executive function network [21, 36, 37]. Whereas automatic walking and rhythmic ST walking stimulate subcortical locomotor regions of the brain, DT walking involves a direct locomotion pathway (i.e., the primary motor cortex, cerebellum, and spinal cord) and an indirect locomotion pathway (i.e., the prefrontal cortex, premotor areas, supplementary motor area, and basal ganglia) [21, 38]. Motor and cognitive therapies are essential components of stroke rehabilitation but are generally performed separately and not simultaneously [16, 24]. Rehabilitation programs based on CMI have shown moderate efficacy in the chronic post-stroke phase [39–42] and in degenerative diseases [42–44]. Randomized controlled trials have assessed the motor and cognitive effects of DT or multitask GR protocols in the chronic post-stroke phase and in other neurological diseases but not at the sub-acute post-stroke stage [28, 39, 44–46]. To the best of our knowledge, this is the first randomized, controlled study of DT GR at the sub-acute stage of stroke. The DT GR program is focused on interactions between motor activity on one hand and five cognitive activities (executive functions, spatial exploration, phasic functions, memory functions, and prehension) on the other.

### Objectives {7}

The primary objective of the trial described here is to determine whether long-term gait velocity is greater after DT GR than after ST GR. The secondary objectives are to compare DT GR and ST GR with regard to motor performance (lower limb motricity, gait, and balance), cognitive performance, personal autonomy, disability, and QoL in the short, medium, and long term after stroke, as a function of the stroke victim's gait characteristics and plasticity after stroke.

### Trial design {8}

SYNCOMOT is a multicenter (*n*=12), prospective, two-arm, parallel-group, randomized [1:1], stratified, controlled superiority trial conducted in France.

## Methods: participants, interventions, and outcomes

### Study setting {9}

**Fig. 1:**
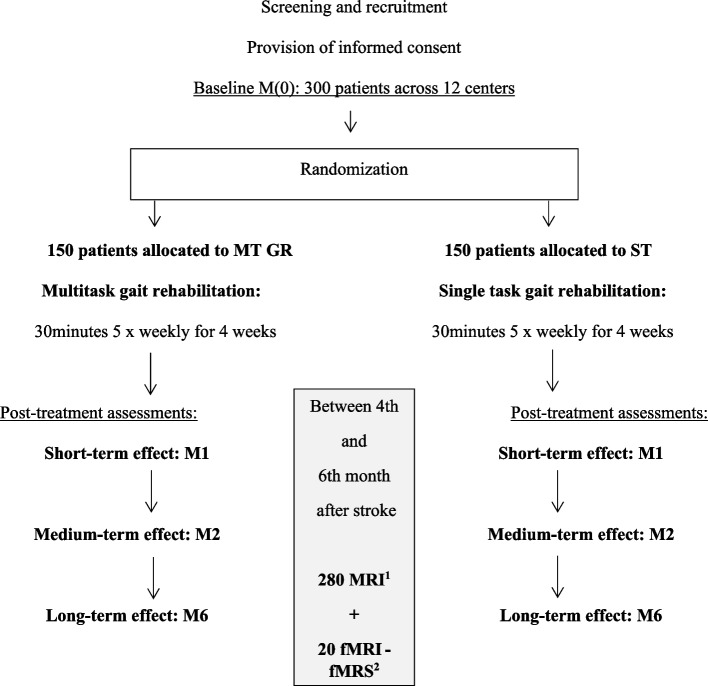
Study design and flow diagram. MRI, magnetic resonance imagery; fMRI, functional MRI; fMRS, functional magnetic resonance spectroscopy

Patients will be recruited by neurorehabilitation departments at 12 clinics, general hospitals, and university medical centers in northern France: Amiens Picardie University Medical Center (the coordinating center, in Amiens), Arras General Hospital (Arras), Beauvais General Hospital (Beauvais), the Centre Jacques Calvé clinic (Berck-sur-Mer), the Institut Medical de Breteuil clinic (Breteuil), Caen University Medical Center (Caen), Compiegne General Hospital (Compiegne), the Centre de Réeducation des Trois Vallées clinic (Corbie), Lille University Medical Center (Lille), the Centre L'Espoir clinic (Lille), the Centre Le Belloy clinic (Saint Omer-en-Chaussée), and Rouen University Medical Center (Rouen). The study flow chart for screening, enrolment (after the provision of written, informed consent), and randomization is shown in Fig. 1.

### Eligibility criteria {10}

The inclusion criteria will be as follows: (i) age between 18 and 90, (ii) admission to a rehabilitation department within 6 months of a first ischemic or hemorrhagic stroke with positive brain imaging findings (computed tomography (CT) or magnetic resonance imaging (MRI) of the brain), (iii) ability to provide informed consent, (iv) ability to walk at least 10 m (with or without a technical aid) at a speed below 1.2 m.s^−1^, and (v) social security coverage. The exclusion criteria will be (i) prestroke locomotor or neurological disorders affecting gait, (ii) visual or hearing disorders that impair communication, (iii) severe aphasia, (iv) participation in another interventional study of gait or cognitive rehabilitation, (v) pregnancy or breastfeeding, and (vi) administrative supervision (legal guardianship or incarceration).

### Who will take informed consent? {26a}

All participants will be fully informed about the trial’s objectives and procedures by rehabilitation specialists (Physical Medicine and Rehabilitation (PM&R) physicians) and will give their written, informed consent to the center’s lead investigator (also a rehabilitation specialist) prior to inclusion. Participants will be informed in the patient information leaflet that the study data will be kept safe.

### Additional consent provisions for collection and use of participant data and biological specimens {26b}

Not applicable.

## Interventions

### Explanation for the choice of comparators {6b}

The DT GR program is focused on interactions between motor activity on one hand and five cognitive activities (executive functions, spatial exploration, phasic functions, memory functions, and prehension) on the other. The trial will compare the short-, medium- and long-term effects of a DT protocol vs. a ST protocol initiated during the sub-acute post-stroke period.

### Intervention description {11a}

GR program is based on a personalized, progressive DT approach; with repetitive, goal-oriented tasks. At each site, at least two trained physiotherapists will deliver the intervention in the two parallel groups. The GR sessions will include 30 min of physical therapy on 3 days a week for 4 weeks after inclusion (i.e., 12 sessions in total), instead of the patient’s standard rehabilitation sessions on those days. Each 30-min session will consist of five different 5-min exercises, with 1 min of rest between each exercise. The GR protocols will focus on ST gait or on cognitive-motor DT gait. The patient’s progression through the protocol will be standardized according to the self-assessed cognitive and motor burdens (rated on visual analog scales (VASs)) at the end of each session. If the cognitive or motor burden of the last session is below 7 on the VAS, the intensity in the next session will be incremented by one level. Ideally, the intensity of the patient’s GR will increase week after week. If the patient fails an exercise or session, it will be replaced by the last exercise or session in which the patient was successful. The sessions’ functional task training will combine intensity with functional relevance [16]. The care providers’ adherence to the rehabilitation protocol will be evaluated during study monitoring visits.

#### The control group: ST GR program (Table 1)

The ST GR program will be composed of 5-min gait exercises in five different walking directions: forwards, sideways on the healthy side, sideways on the hemiparetic side, backwards, and stepping up over obstacles or foam mats). As mentioned above, progression over the 4 weeks will depend on the self-assessed motor burden (VAS score) at the end of each session. The intensity of the ST gait protocol will be increased by adding weight on the hemiparetic side, increasing the gait speed, increasing the height of the obstacles, and increasing balance constraints (having to walk along a line). Depending on the patient's progression through the gait protocol, up to 12 intensity levels can be performed. If a patient fails an exercise or session, she/he will repeat the last successfully completed exercise or session (so as not to waste the 30 min of GR).

**Table 1:** The standardized ST GR protocol

Progression	Five types of 5-min gait exercises (30 min per session, 3 times a week on D1, D2, and D3)	Level of motor difficulty
**Week 1**	- Walking forwards - Walking sideways on the healthy side - Walking sideways on the hemiparetic side - Walking backwards - Walking and crossing flat obstacles	1/ Comfortable walking 2/ Walking with weights (100–250 g) 3/ Walking along a line
	If an exercise is impossible, the patient will repeat the last successfully completed exercise or session (so as not to waste the 30 min of GR).	
**Week 2**	- Walking forwards - Walking sideways on the healthy side - Walking sideways on the hemiparetic side - Walking backwards - Walking and crossing obstacles	4/ Comfortable walking with weights (100–250 g) 5/ Comfortable walking with weights (250–500 g) 6/ Comfortable walking and crossing obstacles <5 cm in height
	If an exercise is impossible, the patient will repeat the last successfully completed exercise or session (so as not to waste the 30 min of GR).	
**Week 3**	- Walking forwards - Walking sideways on the healthy side - Walking sideways on the hemiparetic side - Walking backwards - Walking on a foam carpet	7/ Fast walking 8/ Fast walking with weights (100–250 g) 9/ Fast walking on a foam carpet
	If an exercise is impossible, the patient will repeat the last successfully completed exercise or session (so as not to waste the 30 min of GR).	
**Week 4**	- Walking forwards - Walking sideways on the healthy side - Walking sideways on the hemiparetic side - Walking backwards - Walking on a foam carpet	10/ Fast walking with weights (100–250 g) 11/ Fast walking with weights (250–500 g) 12/ Fast walking and crossing obstacles <10 cm in height
	If an exercise is impossible, the patient will repeat the last successfully completed exercise or session (so as not to waste the 30 min of GR).	

#### The experimental group: DT GR program (Table 2)

The DT GR program will be composed of five different 5-min cognitive-motor DTs. The DT gait exercises will be related to instrumental activities of daily living after a stroke and will involve both internal interfering functions (executive functions, phasic functions, memory, and prehension) or external interfering functions (spatial cognition). The therapist will not encourage the patient to prioritize one task over another, i.e., the patient will be free to favor (or not) one of the two tasks if she/he wishes. The difficulty over 4 weeks will be assessed according to the motor and cognitive burdens during the GR program (self-assessed on VASs at the end of each session). The cognitive difficulty will be based on three types of sound environment: quiet, noisy with the patient’s favorite music (chosen on the YouTube website), or the France Info news radio station. If a DT exercise cannot be completed, it will be replaced by another DT exercise, such as a calculation task or a previously completed task (so as not to waste the 30 min of GR).

**Table 2:** The standardized DT GR protocol

Progression	Five types of 5-min gait exercise (30 min per session, 3 times a week (on days (D)1, D2, and D3)	Level of motor difficulty	Level of cognitive difficulty
**Week 1**	- Walking during an executive function task: go/no-go letters (D1: letter “A,” D2: letter “B,” D3: letter “Z”) - Walking during a memory task: minus -2 span - Walking while holding an object in the healthy hand: a half-full glass of water - Walking during a semantic fluency task (D1 animals, D2 fruits, D3 supermarket articles) - Walking during while looking for targets in the space ahead (D1 objects, D2 images, D3 words)	1/ Comfortable walking 2/ Walking with weights (100–250 g) 3/ Walking along a line	1/ A quiet environment 2/ Music selected by the patient 3/ News radio (France Info)
	If an exercise is impossible, replace it with a calculation (plus 2) and the following starting numbers: 0, 1, 14, 43, 128 (5 × 1 min). If this is impossible, count backwards or forwards, depending on the patient's abilities.		
**Week 2**	- Walking during an executive function task: go/no-go Letters (D1: letter “A,” D2: letter “B,” D3: letter “Z”) - Walking during a memory task: minus −1 span - Walking while holding an object in the healthy hand: a 75%-full glass of water - Walking during a semantic fluency task (D1 kinds of bird, D2 towns, D3 colors) - Walking during while looking for targets on the healthy side (D1 objects, D2 images, D3 words)	4/ Comfortable walking with weights (100–250 g) 5/ Comfortable walking with weights (250–500 g) 6/ Comfortable walking and crossing obstacles <5 cm in height	1/ A quiet environment 2/ Music selected by the patient 3/ News radio (France Info)
	If an exercise is impossible, replace it with a calculation (plus 3) and the following starting numbers: 0, 1, 14, 43, 128 (5 × 1 min). If this is impossible, count backwards or forwards, depending on the patient's abilities.		
**Week 3**	- Walking during an executive function task: go/no-go numbers - Walking during a memory task: minus −2 alphabetic span - Walking while holding an object in the paretic hand: a half-full glass of water - Walking during an alternating semantic fluency task (D1 animals/fruits, D2 fruits/vegetables, D3 fruits/supermarket articles) - Walking while looking for targets on the paretic side (D1 objects, D2 images, D3 words)	7/ Fast walking 8/ Fast walking with weights (100–250 g) 9/ Fast walking on a foam carpet	1/ A quiet environment 2/ Music selected by the patient 3/ News radio (France Info)
	If an exercise is impossible, replace it with a calculation (minus 2) and the following starting numbers: 0, 1, 14, 43, 128 (5 × 1 min). If this is impossible, count backwards or forwards, depending on the patient's abilities.		
**Week 4**	- Walking during an executive function task: go/no-go numbers - Walking during a memory task: minus −1 alphabetic span - Walking while holding an object in the paretic hand: a 75%-full glass of water - Walking during an alternating semantic fluency task (D1 kinds of fish/bird, D2 fruits/towns, D3 colors/furniture - Walking while looking for targets in the space behind (D1 objects, D2 images, D3 words)	10/ Fast walking with weights (100–250 g) 11/ Fast walking with weights (250–500 g) 12/ Fast walking and crossing obstacles <10 cm in height	1/ Quiet environment 2/ Music selected by patient 3/ News radio (France Info)
	If an exercise is impossible, replace it with a calculation (minus 3) and the following starting numbers: 0, 1, 14, 43, 128 (5 × 1 min). If this is impossible, count backwards or forwards, depending on the patient's abilities.		

### *Criteria for discontinuing or modifying allocated interventions {11b}*

In the standardized ST GR protocol, if an exercise is impossible, the patient will repeat the last successfully completed exercise or session (so as not to waste the 30 min of GR) or will perform a calculation (week 1: addition “plus 2,” week 2: addition “plus 3,” week 3: subtraction “minus 2,” and week 4: subtraction “minus 3,” with the following starting numbers: 0, 1, 14, 43, 128 (5 × 1 min)). If this is impossible, the patient will count backwards or forwards, depending on his/her abilities. If the GR protocol is suspended for medical reasons (an acute health problem), the 30-min sessions will be rescheduled so that all 12 are achieved within 31 days. Allocated interventions will not be modified.

### Strategies to improve adherence to interventions {11c}

Included patients will join the SYNCOMOT in-patient rehabilitation program in each participating center. The GR rehabilitation protocols will be gradual and personalized, in order to motivate the participants. Trained therapists will ensure that the patients comply with the GR protocol in each session. The level(s) of difficulty (the motor burden and/or the cognitive burden) during the two GR programs will be self-assessed by the patient on VASs at the end of each session and recorded by the therapist. Depending on the patient's progression through the GR protocol, up to 12 intensity levels can be performed. If a patient fails an exercise or session, she/he will repeat the last successfully completed exercise or session. If an exercise is not possible, an alternative exercise will be provided in the protocol and thus will ensure that the patient performs 30 min of GR. If the GR protocol is suspended because of an acute health problem, the 30-min sessions will be rescheduled so that all twelve 30-min training sessions are completed within 31 days.

### Relevant concomitant care permitted or prohibited during the trial {11d}

All types of care will be permitted during the study, other than non-scheduled DT training.

### Provisions for post-trial care {30}

Not applicable.

### Outcomes {12}

#### Clinical assessments at baseline (Table 3)

The study population's characteristics recorded at baseline (M0) will include demographic variables (age, sex, and handedness), comorbidities (neuro-orthopedic and bladder disorders), the stroke date, the type of stroke (site, side, and ischemic or hemorrhagic), the stroke severity (National Institutes of Health Stroke Scale (NIHSS) score) [47], neurological impairment (the motor part of the Fugl-Meyer Assessment of the lower extremity [54], the French adaptation of the Harmonization Standards Neuropsychological Battery [50, 55, 56] (Table 4), personal autonomy (the functional independence measure (FIM) [51]), disability (a structured interview with a modified Rankin scale score (mRS-SI) (Table 5) [52]); and QoL on a VAS (QoL-VAS). Qualitative and quantitative gait variables will be assessed with a clinical classification of gait disorders (Supplementary material 1), the single-task 10-m walking test (ST 10MWT) [61], and the FIM's “walking” item [51]. Balance will be assessed in the timed up-and-go (TUG) test. In order to assess CMI, four cognitive-motor DTs will be performed during the DT 10MWT and the DT TUG test: a verbal Trail Making Test (executive function) [59], a symbolic praxis test [60], a literal fluency test [50], and a verbal serial number recall task (short-term memory [62]). The data from the cognitive-motor DTs will be recorded every 15 s, so that participants can be compared regardless of their gait speed [63].

**Table 3:** Schedule for the assessment of primary and secondary outcomes

	Recruitment	Inclusion M0	Follow-up visit at M1	Follow-up visit at M2	Follow-up visit at M6
**Investigator**					
Patient information	✓				
Informed consent		✓			
Demographic data^a^		✓			
Randomization		✓			
Gait classification		✓			
Stroke^b^		✓			
NIHSS		✓	✓	✓	✓
Fugl-Meyer (lower limb)^c^		✓	✓	✓	✓
FIM		✓	✓	✓	✓
mRS-SI		✓	✓	✓	✓
QoL-VAS		✓	✓	✓	✓
AEs and SAEs			✓	✓	✓
**Therapists**					
ST 10 MWT		✓	✓	✓	✓
DT 10 MWT		✓	✓	✓	✓
ST TUG		✓	✓	✓	✓
DT TUG		✓	✓	✓	✓
**Neuropsychologist**					
Neuropsychological battery^d^		✓	✓	✓	✓
**Radiologist**					
MRI	✓ 280 patients in 12 centers (4 and 6 months after stroke)				
fMRI and fMRS	✓ 20 patients at the Amiens center (4 and 6 months after stroke)				

^a^Age, sex, handedness, and comorbidities (bladder disorders and neuro-orthopedic complications)

^b^Stroke site, side, and type (ischemic or hemorrhagic)

^c^The motor part of the Fugl-Meyer Assessment of the lower extremity [47]

^d^Assessment of neuropsychological cognitive abilities, using the French adaptation [29] of the Harmonization Standards protocol [48]; assessment of behavioral dysexecutive disorders, using the previously validated Behavioral Dysexecutive Syndrome Inventory) [49]

*NIHSS* National Institutes of Health Stroke Scale, *FIM* the Functional Independence Measure [50], *mRS-SI* a structured interview with a modified Rankin score [51], *QoL-VAS* visual analog scale for quality of life, *AE* adverse event, *SAE* serious adverse event, *ST 10MWT* single-task 10-meter walk test [52, 53], *DT 10MWT* dual-task 10-m walk test, with four cognitive tasks during gait, the verbal Trail-Making Test (executive function), a symbol praxis task, a literal fluency task, and a verbal serial recall task (short-term memory), *ST TUG* single-task timed up-and-go test, *DT TUG* dual-task timed up-and-go test, with four cognitive tasks during gait, the verbal Trail-Making Test (executive function), a symbol praxis task, a literal fluency task, and a verbal serial recall task (short-term memory), *MRI* magnetic resonance imagery, *fMRI* functional MRI, *fMRS* functional magnetic resonance spectroscopy

**Table 4:** The validated French adaptation of the Harmonization Standards Neuropsychological Battery for stroke, for describing cognitive functions at the M0, M1, M2, and M6 visits [48, 55]

SYNCOMOT will assess appropriate cognitive abilities (using the French adaptation [29] of the Harmonization Standards Neuropsychological Battery [48]) and behavioral dysexecutive disorders (using the previously validated Behavioral Dysexecutive Syndrome Inventory (BDSI)) [49]	
- Detecting global cognitive impairments:	
• Mini-Mental State Examination [57]	
- *Detection of the main cognitive disorders:*	
• Phasic: center using scale except Battery for the Evaluation of Lexical Disorders [58]	
• Praxis: Florence Mayeux’s symbolic praxis test [59]	
• Memory: short-term memory with an immediate verbal serial number recall (Baddeley task) and long-term verbal and visual memory	
• Spatial neglect: Albert’s test	
- Description of executive functions:	
• Flexibility of attention: Trail-Making Test parts A and B [48]	
• Dual task performance: Baddeley task [60]	
• Inhibition: Stroop tasks [48]	
• Verbal fluency: literal fluency (“P” at M0, “V” at M1, “R” at M2 and “B” at M6)	
• Processing speed index (PSI): third edition of the Wechsler adult intelligence scale (WAIS-III)	
• Dysexecutive behavior syndrome (BDSI) [49]	
- Detection of mood disorders:	
• Depression assessment on the Montgomery-Asberg Depression Rating Scale (MADRS) [90]	
• Anxiety assessment on the Goldberg scale [91]

**Table 5:** Structured interview of modified Rankin Score (mRS-SI): an English translation of the French-validated mRS-SI

**mRS-SI 5**	**Bedridden: ‘Is the person bedridden?’**	
	- The patient is unable to walk even with another person’s assistance.	**Yes □ No □**
	- If placed in a wheelchair, unable to self-propel effectively.	
	- May frequently be incontinent.	
	- Requires nearly constant care (provided by either a trained or untrained caregiver): someone needs to be available nearly at all times.	
**mRS-SI 4**	**Assistance to walk: ‘Is another person’s assistance essential for walking?’**	
	- Requiring another person’s assistance means needing another person to be always present when walking, including indoors around house or ward, to provide physical help, verbal instruction, or supervision.	**Yes □ No □**
	- Patients who use physical aids to walk (stick, walker) but do not require another person’s help, are NOT rated as requiring assistance to walk.	
	- For patients who use wheelchairs, patient needs another person’s assistance to transfer into and out of chair, but can self-propel effectively without assistance.	
**mRS-SI 3**	**Assistance to look after own affairs**^**1**^**:** “Could the patient live alone for 1 week if he/she absolutely had to?”	
3.1	- Is assistance essential for preparing a simple meal?	**Yes □ No □**
3.2	- Is assistance essential for basic household chores?	**Yes □ No □**
3.3	- Is assistance essential for looking after household expenses and to manage day-today purchases?	**Yes □ No □**
3.4	- Is assistance essential for local travel and transportation?	**Yes □ No □**
3.5	- Is assistance essential for taking medication in correct dosages at correct time (includes preparation in advance, recall and supervision)?	**Yes □ No □**
3.6	- Telephone use: Is assistance essential for look up and dial numbers?	**Yes □ No □**
**mRS-SI 2**	**Limitations in participation in usual duties and activities:** “has there been a change in the person’s ability to work or look after others or participate to leisure activities as compared with prestroke status? This supposes that it is due to the new stroke itself.”	
2.1	- Has the stroke substantially reduced the person’s ability to work or, for a student, to study? (Change from full-time to part-time, change in level of responsibility, or unable to work at all).	**Yes □ No □**
2.2	- Has the new stroke substantially reduced the person’s ability to look after family at home?	**Yes □ No □**
2.3	- Has the new stroke reduced the person’s regular free-time activities^2^?	**Yes □ No □**
2.4	- Is this reduction in activity related to a physical/medical problem other than the stroke? If yes which one? Details:	**Yes □ No □**
**mRS-SI 1**	**Persisting symptoms as a result of the stroke:** “Does the patient have any symptoms resulting from the new stroke?” “Does the person have:”	
	- Difficulty reading or writing?	**Yes □ No □**
	- Difficulty speaking or finding the right word?	**Yes □ No □**
	- Problems with balance or coordination?	**Yes □ No □**
	- Visual problems as a result of the stroke?	**Yes □ No □**
	- Numbness (face, arms, legs, hands, feet)?	**Yes □ No □**
	- Weakness or loss of movement (face, arms, legs, hands, feet)?	**Yes □ No □**
	- Difficulty with swallowing	**Yes □ No □**
	- Sleeping difficulty?	**Yes □ No □**
	- Headaches as a result of stroke?	**Yes □ No □**
	- Otherwise unexplained reduction of activities, anxiety, depressive mood, or sadness repetitive concerns (especially about his/her health or situation)?	**Yes □ No □**
	- Loss of consciousness?	**Yes □ No □**
	- Other symptoms? Details:	**Yes □ No □**

1. Assistance includes physical help, verbal instructions, or supervision by others

2. Social and leisure activities include activities outside the home or at home; Activities outside the home: going to the coffee shop, bar, restaurant, club, church, cinema, visiting friends, going for walks; Activities at home: involving “active” participation including knitting, sewing, painting, games, reading books, home improvements

**Procedure**: Examiners had to read each proposal to the patient and caregiver and when impairment was detected, the corresponding Rankin grade had to be selected. The examiner might also gather data from other sources, such as records, nurse notes, and medical files. The examiner was instructed to rate what the patient actually did. The only exceptions concerned situations where the patient could clearly perform the activity in a fully independent manner but it was not performed for contextual reasons: patients able to work but not returning to work because the stroke occurred just before retirement (early retirement); patient able to perform basic activities but the carer prefers to do them because of time pressure; patient not confronted with specific activities (complex financial activities, transportation, meal preparation) since hospital discharge

#### The primary outcome measure

Gait speed is the “gold standard” measure of gait performance [64]. At the subacute stage of stroke, speed was recently estimated to be 0.34 m.s^−1^, i.e., a 70% decrease [53]. The ST 10MWT will be used to assess gait performance in the DT GR and ST GR groups at post-stroke M6. This test is a key indicator of functional gait performance, the risk of falls, and decline after stroke [40, 41]; it constitutes the gold standard for the provision of quantitative data on gait performance. Performance in the 10MWT is known to be related to community ambulation ability and QoL [10, 53, 63–66].

#### Secondary outcome measures

The secondary outcomes measures will be (i) motor performance (lower limb motricity, gait, and balance), (ii) cognitive performance, (iii) autonomy, (iv) disability, and (v) QoL in the short, medium, and long term after stroke, as a function of gait characteristics and post-stroke plasticity. In order to evaluate short-, medium- and long-term effects, these outcomes will be assessed at M1, M2, and M6, respectively. The classification of the various gait characteristics at baseline will describe the epidemiology of gait disorder phenotypes at the sub-acute stroke stage.

### Participant timeline {13}

The participant timeline is shown in Fig. 1.

### *Sample size {14}*

According to Severinsen et al., the mean ± standard deviation 10MWT speed among stroke victims at the chronic stage is 0.84 ± 0.3 m.s^−1^ [65]. With an alpha coefficient of 0.05 (in two-tailed tests), a power of 80%, and a gait speed standard deviation of 0.3 m.s^−1^, we have calculated that a sample size of 280 patients (140 per group) will be needed to evidence a gait speed increment of 0.1 m.s^−1^ (a magnitude known to be clinically relevant after stroke [66–69]).

### Recruitment {15}

With an expected loss to follow-up rate of 10%, 300 patients (150 per group) will be included. To optimize inclusions, each center will screen inpatients upon admission to the stroke rehabilitation unit. Enrollment will continue until the recruitment target is reached (25 patients/site). We acknowledge that because of the COVID-19 pandemic, continuous inclusion will not be possible; hence, the enrollment period will extend over 6 years.

## Assignment of interventions: allocation

### Sequence generation {16a}

The randomization procedure will be prepared by a data manager in the Clinical Research and Innovation Directorate (*Direction de la Recherche Clinique et de l'Innovation*, DRCI) at Amiens Picardie University Medical Center and implemented using Ennov Clinical® electronic data capture software (version 8.0.140, Ennov, Paris, France). The participants will be randomized (1:1) after inclusion and the baseline screening assessments at M0. To obtain groups that are balanced with regard to the stroke prognosis, stroke severity, and center, participants will be stratified by age (<45, 45 to 64, and ≥65), severity (according to the NIHSS score: < 5, 6 to 24, and ≥25), and center (1 to 12).

### Concealment mechanism {16b}

The treating therapist will be notified by the DRCI (by e-mail) of the treatment allocation. The study database will be pseudo-anonymized (with assignment of patient codes) and held on a server with a secure sockets layer certificate. The two GR programs will be applied during 30-min sessions comprising five 5-min exercises, with a 1-min break between exercises (Tables 1 and 2).

### Implementation {16c}

An investigator at each center will enroll participants, and trained therapists (physiotherapists, occupational therapists, and/or sports therapists) will assign participants to interventions.

## Assignment of interventions: blinding

### Who will be blinded {17a}

SYNCOMOT is an open trial; participants and therapists will therefore not be blinded to the intervention allocation. The assessors of the primary and secondary outcomes will not be blinded.

### Procedure for unblinding if needed {17b}

Not applicable.

## Data collection and management

### Plans for assessment and collection of outcomes {18a}

#### The primary outcome measure

The ST 10MWT will be used to compare gait performance in the DT GR and ST GR groups at post-stroke M6. As a single-task assessment of comfortable walking speed during a 10-m walking test, the ST 10MTW is the “gold standard” short-distance test of walking performance in people with stroke [64, 70]. Gait speed is an important determinant of various health aspects, such as vital status, gait ability (balance, mobility limitations, activities of daily living, etc.), and community activities after stroke [11, 61, 67, 71–73].

The ST 10MWT’s psychometric properties are excellent, with strong reliability, construct validity, sensitivity to change across the care continuum after stroke, and excellent clinical test-retest reliability (intraclass correlation coefficients: 0.80–0.99; measurement error: 0.04–0.40) [64]. Gait speed is clinically meaningful and is correlated with measures of strength, balance, and physical activity (*r* = 0.26–0.8, *p* < 0.05) [64]. Each center will receive a training visit and will be given guidelines on performing the ST 10MWT.

#### Secondary outcome measures

The secondary outcomes measures will be (i) motor performance (lower limb motricity, gait, and balance), (ii) cognitive performance, (iii) autonomy, (iv) disability, and (v) QoL in the short, medium, and long terms after stroke, as a function of gait characteristics. In order to evaluate short-, medium- and long-term effects, these outcomes will be assessed at M1, M2, and M6, respectively.

Pre-study training visits will ensure that each center's personnel are trained in the study requirements, standardized measurement, counseling for adherence, and the collection of information from the study participants in a uniform, reproducible manner. The data will be collected in an electronic case report form (e-CRF), and the procedures to be implemented at each visit will be reviewed in detail in the SYNCOMOT manual. Each of the data collection forms and the nature of the required information will be discussed in detail on an item-by-item basis. Entering data forms, responding to data discrepancy queries, and obtaining research-quality data will also be covered during the training session.

The study manual will include details of SYNCOMOT's procedures and the equipment used, in order to standardize clinical practice during the assessments at M0, M1, M2, and M6. Manuals and monitoring visits will increase the data quality and ensure that the centers will not deviate from the protocol.

Data from the e-CRF will be securely transmitted and quality controlled in the same manner as the core data from the coordinating center. Ennov Clinical® data entry software will instantly identify any missing or aberrant data, so that the latter can be completed or corrected as soon as possible.

### Plans to promote participant retention and complete follow-up {18b}

SYNCOMOT is an in-patient protocol; retention is therefore expected to be high because (i) the included patients will be hospitalized in a stroke rehabilitation unit during their GR, and (ii) follow-up at M6 is a routine clinical procedure after stroke. Any deviation from the study protocols will be reported in the e-CRF by training therapists. The Ennov Clinical® data entry software will instantly identify any missing or aberrant data, so that the latter can be completed or corrected as soon as possible. This multicenter, randomized, controlled superiority trial will compare DT versus ST GR programs for all allocated patients with available data, on an intention-to-treat basis.

### Data management {19}

Therapists will collect data on paper CRFs and will then enter the data into an Ennov Clinical® e-CRF. Data quality will be enhanced by the application of Ennov Clinical® data capture software and monitoring visits (with checks on data entry, coding, security, and storage).

### Confidentiality {27}

The study database will be pseudo-anonymized (with assignment of patient codes) and held on a server with a secure sockets layer certificate, in order to protect confidentiality before, during, and after the trial. The patient-information leaflet will state that the study data will be stored for 15 years. The personal data will be accessible only to the coordinating investigator, the relevant health authorities (for official inspections), or representatives duly appointed by the sponsor (for quality audits).

### Plans for collection, laboratory evaluation, and storage of biological specimens for genetic or molecular analysis in this trial/future use {33}

Not applicable.

## Statistical methods

### Statistical methods for primary and secondary outcomes {20a}

#### Primary outcome measure

The primary outcome measure will be the ST 10MWT result 5 months after the end of the GR protocol. Depending on the type of data and after adjustment for the patient’s age, center and the NIHSS score in an analysis of covariance (ANCOVA), an independent *t*-test, a Mann-Whitney test, or a generalized linear mixed model will be used to compare the two groups with regard to the 10MWT speed.

#### Secondary outcome measures

After adjustment for the patient’s age, center, and NIHSS score (in an ANCOVA), an independent *t*-test or a Mann-Whitney test will be used to assess intergroup differences in the secondary outcomes: motor skills (including lower limb motricity: the motor part of the Fugl-Meyer Assessment of the lower extremity), gait (10MWT), ambulation capacity (FIM) and balance (TUG)), cognitive skills (neuropsychological assessments of the French validated battery for stroke), personal autonomy (FIM), disability (mRS-SI), and QoL-VAS. As appropriate, Pearson’s correlation coefficient or Spearman's correlation coefficient (r) will be used to assess performance in gait and balance DTs (eight cognitive-motor DTs: phasic, executive, praxis, memory DT 10MWT and DT TUG tasks) and (in a univariate analysis) to identify cognitive and motor determinants correlated with an increment in ST 10MWT speed. We shall rule out multicollinearity by applying standard procedures. Multiple linear regression will then be used to identify factors that are independently correlated with an increment in gait speed.

Between-group comparisons at baseline will be performed using a t-test, a Mann-Whitney test, a Kruskal–Wallis test, a chi-squared test, or Fisher’s exact test, as appropriate. Statistically significant group interactions and factors will be analyzed post hoc. The Bonferroni method will be used to adjust the overall level of significance for multiple testing. All statistical analyses will be performed using SAS software (version 9.3, SAS institute Inc, Cary, NC, USA). The threshold for statistical significance will be set to *p*<0.05. A statistician blinded to the study group assignment will perform the statistical analyses.

### Interim analyses {21b}

Not applicable.

### Methods for additional analyses (e.g., subgroup analyses) {20b}

Not applicable.

### Methods in analysis to handle protocol non-adherence and any statistical methods to handle missing data {20c}

This multicenter, randomized, controlled superiority trial will compare DT versus ST GR for all allocated patients with available data, on an intention-to-treat basis. A generalized linear mixed model could be used to compare the two groups with regard to the 10MWT speed or secondary outcomes.

### Plans to give access to the full protocol, participant level-data and statistical code {31c}

SYNCOMOT’s full protocol (Tables 1, 2, and 3) and datasets during and/or analyzed during the current study are available from the corresponding author on reasonable request. The participant-level dataset will be accessible only to the coordinating investigator, the relevant health authorities (for official inspections), or representatives duly appointed by the sponsor (for quality audits) via an individual, secure online connection (personal login and password) via Ennov Clinical® software. The datasets analyzed during the present study will be available from the corresponding author on reasonable request.

## Oversight and monitoring

### Composition of the coordinating center and trial steering committee {5d}

The study is sponsored by Amiens Picardie University Medical Center. The coordinating investigator is the head of the medical center’s neurological rehabilitation department and will train the investigators at the other investigating centers. The DRCI will host the study’s steering committee (comprising a clinical research associate, a methodologist, a data manager, a biostatistician, a physical medicine and rehabilitation physician, and a project manager). The coordinating investigator (a physical medicine and rehabilitation physician) and the DRCI will provide day-to-day organizational support and study monitoring. The steering committee represented by clinical research associate and coordinating investigator will meet at the beginning and the end of the trial at each center and on request as needed. Implementation and monitoring visits at all investigating centers will be performed as required, and inclusion curves will be circulated as needed.

### Composition of the data monitoring committee, its role and reporting structure {21a}

The study data monitoring committee will comprise a clinical project manager, a data manager, and a clinical research associate from the DRCI, none of whom have conflicts of interest. The funding body has no role in the study design or the collection, analysis, interpretation, and presentation of the study data.

### Adverse event reporting and harms {22}

Adverse events might include falls, fatigue, headache, or other medical events after inclusion. All adverse events (even those not necessarily associated with the intervention) will be recorded on the participant's individual electronic case report form at each study visit. A safety report will be sent to the pharmacovigilance unit and the study sponsor once a year. Serious adverse events will be reported to the study sponsor within 48 h; the latter will then inform all the investigating centers.

### Frequency and plans for auditing trial conduct {23}

The trial will be audited by the DRCI at least once during the study and then at the end of the study. Monitoring and auditing will be independent of the coordinating investigator and investigating centers.

### Plans for communicating important protocol amendments to relevant parties (e.g., trial participants, ethical committees) {25}

All protocol modifications approved by the institutional review board will be communicated to the concerned parties and investigators.

### Dissemination plans {31a}

The coordinating investigator will publish study results via scientific and medical publications. An appropriate newsletter will be sent to participants, the public, and other non-professional groups.

## Discussion

The resumption of independent ambulation and executive functioning is a major independent factor in post-stroke disability and is therefore a priority for rehabilitation [7]. SYNCOMOT is relevant because it will assess the short-, medium- and long-term efficacy of a standardized, randomized, controlled DT GR program. This GR trial will be one of the first to assess cognitive-motor multitasking at the subacute stroke stage [74]. DT and multitask rehabilitation programs are known to be of value in neurodegenerative diseases and in chronic-stage stroke [39, 40, 44, 45]. However, to stimulate perilesional functional cerebral networks of value in everyday living, neural plasticity needs to be guided from the sub-acute stage onwards [75]. One of the advantages of multitask rehabilitation is the combination of physical gait exercises, which increases the perfusion of the heart and the brain during cognitive tasks. Cognitive functioning during hemodynamic stimulation might be synergistic (thanks to the synthesis of neurotrophic factors) and might stimulate the brain's functional plasticity [76, 77].

This study has several complementary strengths. Firstly, the rehabilitation protocols have been designed to increase the degree of synergy between cognitive and motor functions and thus enhance all aspects of the International Classification of Functioning, Disability, and Health core set for stroke (i.e., gait, personal autonomy, social participation, and QoL). This personalized, standardized rehabilitation protocol for gait, balance, and cognitive skills will mix cognitive tasks involving internal interfering factors (via phasic, praxis, executive function, and memory tasks) and tasks involving external interfering factors (spatial cognition) and thus will call upon both low-level and high-level cognitive systems. Moreover, the GR program will use concomitant motor tasks (e.g., a praxis DT with hand engagement) known to have a greater impact on walking, along with complex cognitive tasks involving executive function during gait (e.g., an executive DT with a go/no-go task) [78]. Gait and cognitive performance will be assessed with gold-standard STs: the 10MWT [64] and the French adaptation of the Harmonization Standards Neuropsychological Battery [56]. The same care providers will deliver the intervention in both groups. Our robust assessment of CMI via four complementary cognitive tasks (phasic, praxis, memory, and executive function tasks) and two types of motor activity (gait and dynamic balance) should provide more details about these aspects [79].

The study’s main limitation is its open design: the protocol will not be blinded to the patients or therapists. Secondly, the primary outcome is performance in an ST (gait in the 10 MWT), rather than in a DT. We chose to use the 10 MWT because it is the gold standard for gait performance and is linked to a decline in the elderly [80, 81], post-stroke social inclusion [82], and mortality [11, 64, 83]. After stroke, patients prioritize gait autonomy in their rehabilitation goals [84]. Furthermore, we have found that gait disorders (rather than behavioral and cognitive disorders) are among the main determinants of disability at post-stroke M6 [7]. Although we could have chosen cognitive function as the study's primary criterion, we preferred to focus on walking speed as a marker of walking function [67] but also of survival [11], motor and balance functions, autonomy via community ambulation [85], social participation, and health-related QoL [9, 83, 86].

The study's future results might open up perspectives in the field of rehabilitation. The cognitive effects of DT rehabilitation might be of value for patients because cognitive impairment is one of the main determinants of post stroke disability [7, 87]. It has been estimated that respectively two thirds and one third of patients have subjective memory complaints and objective memory impairments 21 months post-stroke [48, 88]. These cognitive impairments have a negative effect on the patient’s functional independence [49]. The long-term efficacy of cognitive rehabilitation protocols is subject to debate, even though this question has been extensively studied [24, 57, 58]. Assessment of the relationship between the patients’ gait classification and post-stroke impairments might open up other perspectives. This classification identifies quantitative and qualitative differences according to the phenotype of gait and might help to target the GR program on specific disorders. We hope that a structural and functional neuro-imaging analysis of post-stroke plasticity will help us to better understand the neuronal networks involved in motor and cognitive recovery. Although researchers have applied the capacity-sharing, bottleneck, and cross-talk theories to CMI and its neural correlates in humans, further studies are needed [33]. Our findings are likely to be transferable to the clinic because a DT GR program could be adapted for easy, routine use with inpatients and outpatients after stroke or with other neurologic diseases.

This novel study of the sub-acute post-stroke period onwards is intended to help patients recover ecological gait skills; independent walking is essential for activities of daily living and for social inclusion. The ability to engage in a concurrent cognitive task while walking is impaired after stroke and needs specific rehabilitation. A DT GR program might increase the interaction between cognitive and motor functions and thus improve personal autonomy, social participation, and QoL. The interaction between cognitive function and gait is a fascinating area of research with practical clinical implications in neurological diseases and aging. Independent living in the community is important for decreasing the stroke burden for patients and for society.

## Trial status

Version five of the protocol was issued on December 9, 2021. Inclusion began on March 17, 2017, and recruitment should be complete in 2023. Recruitment was suspended during the coronavirus disease 2019 pandemic. We hope to open new investigating centers (for example, Le Havre University Medical Center (Le Havre) is the last center to have been recruited in 2022) and thus increase the number of participants.

## Supplementary Information

**Additional file 1: Supplementary material. **classification of gait disorders after stroke, according to a qualitative gait analysis.

## Data Availability

The datasets during and/or analyzed during the current study are available from the corresponding author on reasonable request. Individual data will be accessible only to the coordinating investigator, or to the relevant health authorities for official inspection or a quality audit by official representatives appointed by the sponsor.

## Abbreviations

10MWT: 10-m walking test
ANCOVA: Analysis of covariance
CI: Confidence interval
CMI: Cognitive-motor interference
CRF: Case report form
CT: Computed tomography
DRCI: Clinical Research and Innovation Directorate (*Direction de la Recherche Clinique et de l'Innovation*)
DT: Cognitive-motor dual-task
e-CRF: Electronic case report form
FIM: Functional independence measure
MRI: Magnetic resonance imaging
fMRS: Functional magnetic resonance spectroscopy
fMRI: Functional magnetic resonance imaging
GR: Gait rehabilitation
M0: Baseline
mRS-SI: Structured interview for the modified Rankin scale score
MRI: Magnetic resonance imaging
NIHSS: National Institutes of Health Stroke Scale
QoL: Quality of life
QoL-VAS: Quality of life on a visual analog scale
ST: Single-task
SYNCOMOT: “SYNergie Cognitivo-MOTrice” in French, which means “cognitive motor synergy” in English
TUG: Timed up-and-go
VAS: Visual analog scale
