# Supplementary material for: Dual-task versus single-task gait rehabilitation after stroke: the protocol of the cognitive-motor synergy multicenter, randomized, controlled superiority trial (SYNCOMOT)
Source: Trials. 2023 Mar 8;24:172. doi: 10.1186/s13063-023-07138-x (PMC9994785; doi:10.1186/s13063-023-07138-x)
Supplement: Supplementary file 1 — Additional file 1: Supplementary material. classification of gait disorders after stroke, according to a qualitative gait analysis. [file 13063_2023_7138_MOESM1_ESM.docx]

**Classification of gait disorders after stroke, according to a qualitative gait analysis**

- Paretic gait disturbances: hemiparesis with foot drop (weakness of foot dorsiflexion control), knee release or recurvatum, weakness of knee control … (e.g., steppage due to peroneal nerve paralysis)
- Spastic gait disturbances: stiff gait with spasticity predominant over lower limb extensors (e.g., hip circumduction due to varus equinus and stiff knee gait of hemiparetic stroke patients)
- Ataxic gait: slam the foot hard onto the ground, exacerbation when patients cannot see their feet. Difficulty walking in a straight line, lateral veering, poor balance, a widened base of support, inconsistent arm motion, and lack of repeatability. (e.g., sensory and cerebellar gait disturbances)
- Gait apraxia: in the weight‐bearing position, the initiation and the execution of the lower‐limb motor activity required for walking is markedly impaired without motor weakness, sensory loss or cerebellar dysfunction in the lower limbs to account for the gait difficulty (e.g., gait of Parkinson syndrom).
- Antalgic gait disturbance: disruption in a person’s walking pattern that’s usually caused by pain. In an antalgic gait, the phase when you stand is shorter than when you swing the other leg forward to take the next step (e.g., limp of arthritis)
- Hypokinetic gait: freezing of gait, walking with slow little steps, festination, slow turning, reduced arm swing (e.g., gait of extrapyramidal disease)
- Hyperkinetic gait: irregular, jerky, involuntary movements in all extremities interfering with gait broad base variable stride length and cadence (e.g., gait of dyskinesia, chorea, athetosis)
- Dual task gait disturbances: walking is disturbed when it is distracted by a second task but walking is normal in single task (e.g., stop walking when talking).
- Major postural instability: anteroposterior and lateromedial trunk instability during standing and walking (e.g., pusher syndrome)
- Anxiety-related: none neurological gait disturbance but just the fear of falling. Walking is easier when it is distracted by a second task (e.g., fear of falling).
- Unclassified gait: gait disorder does not fit previous categories.

*SYNCOMOT will describe the different kinds of gait classification after stroke, as the percentage [95% confidence interval]. In order to better understand the gait disorder classification data, each gait category will be assessed with regard to the patient's clinical and MRI characteristics, gait, balance, and cognitive skills.*
